# Immunization against inhibin DNA vaccine as an alternative therapeutic for improving follicle development and reproductive performance in beef cattle

**DOI:** 10.3389/fendo.2023.1275022

**Published:** 2024-02-15

**Authors:** Jinzhu Meng, Qiuye Li, Lilin Xiao, Weichen Liu, Zhengjie Gao, Lin Gong, Xianyong Lan, Shuilian Wang

**Affiliations:** ^1^ College of Veterinary Medicine, Hunan Agricultural University, Changsha, China; ^2^ Guizhou Provincial Key Laboratory for Biodiversity Conservation and Utilization in the Fanjing Mountain Region, Tongren University, Tongren, China; ^3^ College of Animal Science and Technology, Northwest Agriculture and Forestry University, Yangling, China

**Keywords:** inhibin, vaccine, immunization, reproductive performance, beef cattle

## Abstract

The objective of the present study was to investigate the potential role of immunization against INH on follicular development, serum reproductive hormone (FSH, E_2_, and P_4_) concentrations, and reproductive performance in beef cattle. A total of 196 non-lactating female beef cattle (4-5 years old) with identical calving records (3 records) were immunized with 0.5, 1.0, 1.5, or 2.0 mg [(T1, n = 58), (T2, n = 46), (T3, n = 42) and (T4, n = 36), respectively] of the pcISI plasmid. The control (C) group (n = 14) was immunized with 1.0 mL 0.9% saline. At 21d after primary immunization, all beef cattle were boosted with half of the primary immunization dose. On day 10 after primary immunization, the beef cattle immunized with INH DNA vaccine evidently induced anti-INH antibody except for the T1 group. The T3 group had the greatest P/N value peak among all the groups. The anti-INH antibody positive rates in T2, T3 and T4 groups were significantly higher than that in C and T1 groups. RIA results indicated that serum FSH concentration in T2 group increased markedly on day 45 after booster immunization; the E_2_ amount in T3 group was significantly increased on day 10 after primary immunization, and the levels of E_2_ also improved in T2 and T3 groups after booster immunization; the P_4_ concentration in T2 group was significantly improved on day 21 after primary immunization. Ultrasonography results revealed that the follicles with different diameter sizes were increased, meanwhile, the diameter and growth speed of ovulatory follicle were significantly increased. Furthermore, the rates of estrous, ovulation, conception, and twinning rate were also significantly enhanced. These findings clearly illustrated that INH DNA vaccine was capable of promoting the follicle development, thereby improving the behavioral of estrous and ovulation, eventually leading to an augment in the conception rates and twinning rate of beef cattle.

## 1 Introduction

In the next decades, beef production needs to increase by 120% to feed a growing world population which was estimated by United Nations (1). The multiple ovulation and embryo transfer (MOET) technique has contributed to accelerate calf production. However, the variability in multiple ovulation response of embryo transfer protocols makes it not easy to obtain a suitable number of viable embryos from donors with high genetic potential at a given time (2). In addition, it has been reported that the repeated use of equine chorionic gonadotropins (eCGs) to induce superovulation is capable of leading to ovarian refractoriness and have clear negative effects on reproduction (3, 4).

Inhibins (INH) plays a vital role on the hypothalamus-pituitary gonadal (HPG) axis, which is secreted by sertoli cells of testis in males and granulosa cells of ovarian follicles in females (5, 6). INH has been proposed as an autocrine/paracrine factor that regulates follicular atresia, growth, steroidogenesis and gonadotropin responsiveness (7, 8). High concentrations of INH can exert negative impact on oocyte quality and embryo development by affecting follicle development (9, 10). Hence, passive and active immunizations against INH were performed to develop alternative methods for superovulation in domestic animals.

In the previous study, neutralization of endogenous INH through active immunization could increase the ovulation rate in several mammalian species, including sheep (11, 12), gilts (13), goats (14), and beef cattle (4). In buffaloes, immunization with AMH-INH-RFRP DNA vaccine could improve follicle development and fertility (15). Yet, the effects of active immunization against INH DNA vaccines on reproductive functions of beef cattle are not completely understood. Here, the current study aimed to investigate the potential role of gene immunization against INH on immune responses, follicle development, serum reproductive hormone (FSH, E_2_, and P_4_) concentrations, and evaluate the reproductive effect of this vaccine on estrous, ovulation, and conception rates in beef cattle. Our findings will provide new insights for exploring INH gene immunization technology to induce twins in beef cattle.

## 2 Materials and methods

### 2.1 Preparation of pCISI DNA vaccine

pCISI DNA vaccine has been constructed and preserved in our laboratory previously. Briefly, the synthesized porcine inhibin (INH) α (1–32) (NM_214189) fragment with NdeI and HindIII recognition sites at 5’ and 3’ ends was introduced into the S gene 3’ end of HBsAg-S and the resulting plasmid was named pCIS. Subsequently, PCR was performed to amplify the expected INH gene fragment (114 bp) from the pCIS plasmid, and the products were digested using BamHI. The INH fragment with BamHI recognition site at both ends was incorporated between 112 and 113 amino acid in the coding region of S gene of pCIS to construct the recombinant plasmid pCISI encoding HBsAg-S and two copies of INH fragments. Finally, pCISI plasmid was transformed into *Escherichia coli* (*E. coli*) DH5α producing pCISI DNA vaccine delivered by *E. coli*.

*E. coli* containing pCISI plasmid was inoculated in Luria-Bertani (LB) solid medium with ampicillin and cultured at 37°Cfor 16 h. Then, a single selected colony was randomly picked and added to LB liquid medium (5mL) with ampicillin (60 µg/mL) and cultured in 37°C shaker at 180 rpm/min for 6 h. The *E. coli* solution was transferred (1: 500) to LB liquid medium in large bottles and cultured in 37°C shaker at 180 rpm/min for 10 h. After harvesting, the DNA vaccines were extracted using EndoFree Maxi Plasmid Kit (Tiangen, Beijing, China) according to the manufacturer’s instructions.

### 2.2 Animals and vaccination

All animal experimental procedures were performed in accordance with the National Institutes of Health (NIH) Guidelines and the guide for the Chinese Association for Laboratory Animal Science. All cattle were treated according to the Ethical Committee of Animal Experiments, Hunan Agricultural University (No. 432022021). Nanyang cattle used in this study were raised under the same conditions at Tanghe Animal Husbandry Co. LTD. (Tanghe, China). A total of 196 non-lactating female beef cattle (4-5 years old) with identical calving records (3 records) were selected and divided into five groups: groups T1 (0.5 mg, n = 58), T2 (1.0 mg, n = 46), T3 (1.5 mg, n = 42) and T4 (2.0 mg, n = 36) which were intramuscular injection immunized at both the left and right buttock, respectively. The control (C) group (n = 14) was intramuscular injection immunized at both the left and right buttock with 1.0 mL 0.9% saline. At 21d after primary immunization, all beef cattle were boosted with half of the primary immunization dose. Blood was collected from the jugular vein on day 10 and 21 after primary immunization, on day 10 and 45 after booster immunization. Then, the blood samples were centrifuged at 3000 rpm under 4°Cfor 15 min to separate the serum, which were stored at -80°C for further analysis.

### 2.3 Synchronous estrus and ultrasonography detection

Estrus was synchronized in all the experimental Nanyang beef cattle with two injections of prostaglandin F2α (PGF2α; Prostamate; IVX Animal Health, MO, USA) administered 14 days apart, and the follicles with different diameter sizes (large follicles: ≥10mm in diameter; medium follicles: 7-9 mm in diameter; small follicles: 5-7 mm in diameter) were observed and recorded by daily ultrasonography. According to the records of daily ultrasonography, the growth speed of ovulatory follicles was analyzed. Oestrus was determined by the mounting and the obvious vaginal mucous discharge of beef cattle twice a day. Ovulation was confirmed by the abrupt disappearance of the previously detected dominant follicle on daily ultrasonography. Pregnancy diagnosis was performed on day 35 after artificial insemination (AI) to evaluate the number of embryos and twin rate.

### 2.4 Detection of antibodies against INH in serum

Specific IgG antibodies of serum were determined using an indirect ELISA with synthetic INHα (1-32) antigens (Sigma, USA) as standard antigens on day 10 and 21 after primary immunization, on day 10 and 21 after booster immunization. Briefly, 96-well ELISA plates (Gibco BRL, USA) were coated with 100 ng of standard antigen and kept at 4°C for 12 h. Then, the plate was washed with PBST (PBS with 0.1% Tween-20 (Bioroxx, Germany)) for 6 times, 200 μL of 1% (w/v) BSA in PBST was added into each well and blocked at 37°Cfor 1 h after discarding the reaction reagent. Next, 100 μL of serum samples (diluted to 1:50 with PBST) was added into each well and the plate was incubated at 37°C for 1 h. The bound antibody was detected by adding a second antibody (rabbit anti-bovine) IgG-HRP (diluted to 1:2000 with PBST, Sigma, USA) after washing 6 times with PBST, and then incubated at 37°Cfor 1 h. Finally, 150 μL of tetramethylbenzidine was added into each well and incubated at 37°C. After 30 min, the reaction was stopped with 2 M H_2_SO4 (50 μL/well), and the optical density was measured at 450 nm using a microplate reader (Bio-Rad, USA). ELISA results were analyzed in terms of P/N ratios, where P was used to denote the OD value of the test blood samples, and N was denoted the OD value of the negative control blood samples. The P/N ratios greater than 2.0, and OD values greater than 0.2 were considered positive responses (16).

### 2.5 Hormone determination in serum

Serum concentrations of E_2_, P_4_ and FSH were measured by radio-immunoassay (RIA) using commercial kits (Furui Bioengineering, China). The assay sensitivity and intra-assay CVs were 0.4 IU/L and 2.9% for FSH, 0.5 pg/mL and 7.3% for E_2_, 0.02 ng/mL and 8.8% for P_4_. All samples were measured in thrice.

### 2.6 Statistical analysis

All data were presented as means ± SEM and analyzed using SPSS Statistics 26.0 software (SPSS Software, Inc., Chicago, USA). Data was analyzed using one-way analysis of variance (ANOVA) test followed by Dunnett’s multiple comparison test and significant difference was inferred for *p* < 0.05 and extremely significant difference for *p* < 0.01.

## 3 Results

### 3.1 Detection of antibody in serum of beef cattle

**Figure 1:**
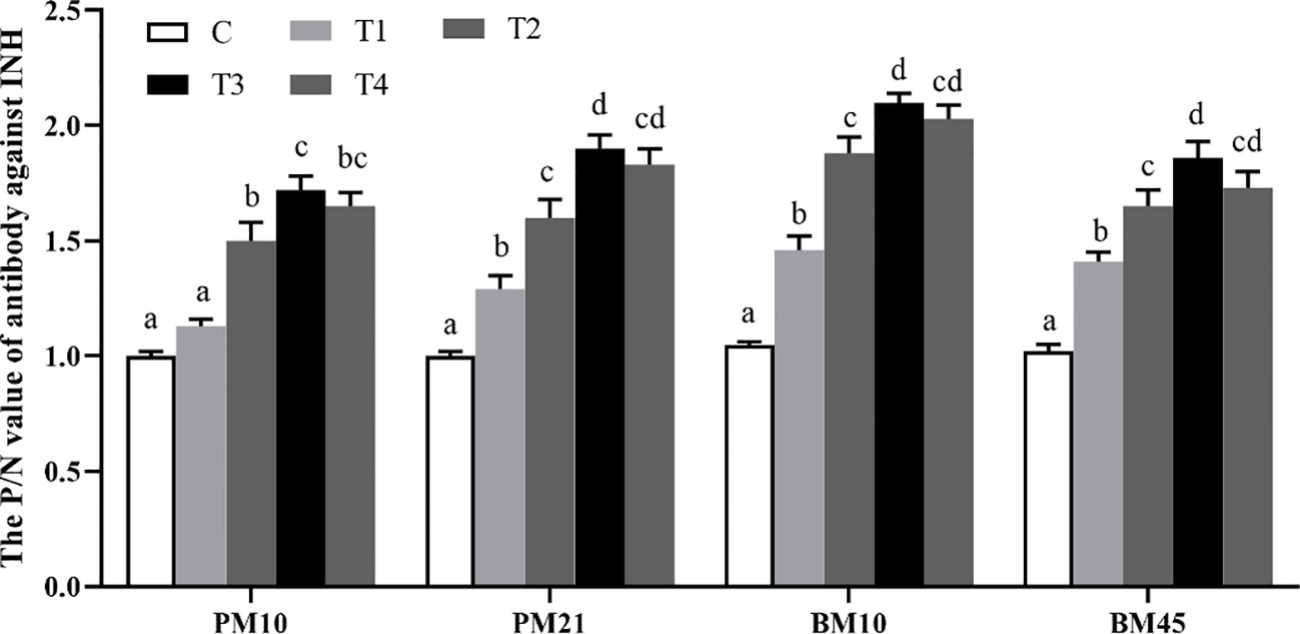
The P/N value of each dosage-group after INH gene immunization. Data are presented as mean ± SEM. The bars with different lower case letters indicate significant differences *p* < 0.05 among groups.

As shown in **Figure 1**, the beef cattle immunized with INH DNA vaccine evidently induced anti-INH antibody, except for the T1 group on day 10 after primary immunization (PM10) compared with the C group. T3 group showed remarkably greater anti-INH antibody amounts than T1 and T2 groups (*p* < 0.05) on day 10, 21 after primary immunization (PM10, PM21) and on day 10, 45 after booster immunization (BM10, BM45).

Furthermore, the positive rates of INH antibodies were calculated in each dosage group after primary immunization and booster immunization (**Table 1**). The anti-INH antibody positive rates in T2, T3, and T4 groups were significantly higher than those in C and T1 groups (*p* < 0.01 or *p* < 0.05) on day 10 and 21 after primary immunization. After booster immunization, the antibody positive rates in T2, T3, and T4 groups significantly increased compared to C and T1 groups (*p* < 0.01). Especially, there was a sharply increase in T3 group of the antibody positive rates compared to other groups (*p* < 0.05) after the booster immunization.

**Table 1:** Percentage (proportion) of the positive antibody after immunization with different vaccine concentrations.

Group	Primary immunization		Booster immunization	
	PM10	PM21	BM10	BM45
C	0%(0/14) ^a^	0%(0/14) ^Aa^	0%(0/14)^Aa^	0%(0/14) ^Aa^
T1	0%(0/58) ^a^	8.59%(5/58)^Aa^	15.51%(9/58) ^Aa^	3.45%(2/58) ^Aa^
T2	17.39%(8/46)^b^	21.74%(10/46)^Ab^	65.22%(30/46) ^Bb^	30.43(14/46)^Bb^
T3	28.57%(12/42)^b^	52.38%(22/42)^Bc^	90.48%(38/42) ^Bc^	52.38%(22/42)^Bc^
T4	22.22%(8/36)^b^	50.00%(18/36)^Bc^	72.22%(26/36) ^Bb^	38.89%(14/36)^Bb^

The different superscript capital and lower case letters in the same column indicate a significant difference p<0.01 and p<0.05 respectively among groups.

### 3.2 Serum hormone concentrations in beef cattle

**Figure 2:**
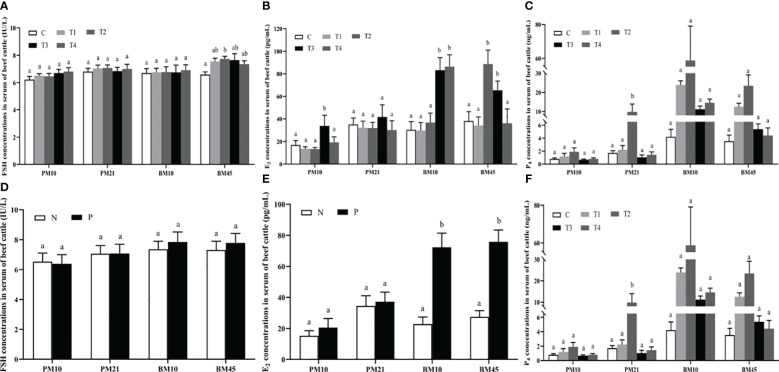
The effect of INH DNA vaccine on FSH **(A, D)**, E_2_ **(B, E)**, and P_4_ **(C, F)** levels in serum of beef cattle. Data are presented as mean ± SEM. The bars with different lower case letters indicate significant differences *p* < 0.05 among groups.

On day 45 after booster immunization, serum FSH concentration in T2 group increased markedly compared with C group (*p*<0.05); no significant difference was observed among all groups for FSH amounts on day 10, 21 after primary immunization and on day 10 after booster immunization (*p*>0.05) (**Figure 2A**). Compared with the C group, the E_2_ amount in T3 group was significantly increased by 20.26 pg/mL (*p*<0.05) on day 10 after primary immunization; levels of E_2_ also improved in T2 and T3 groups compared with the C group (*p*<0.05) on day 10 and 45 after booster immunization (**Figure 2B**). For the P_4_ concentration in serum, we observed that T2 group was significantly greater than C group (*p*<0.05) on day 21 after primary immunization (**Figure 2C**). In addition, we compared the differences of serum FSH, E_2_, and P_4_ amounts between the antibody-positive (P) and antibody-negative (N) beef cattle. The results revealed that there was no significant difference in the amount of FSH among groups (*p*>0.05) (**Figure 2D**). The concentrations of E_2_ in P group were significantly increased compared with those in N group of beef cattle after booster immunization (*p*<0.05) (**Figure 2E**). The concentrations of P_4_ in P group were significantly higher than those in N group of beef cattle on day 45 after booster immunization (*p*<0.05) (**Figure 2F**).

### 3.3 Effect of INH DNA vaccine on follicular development in beef cattle

**Figure 3:**
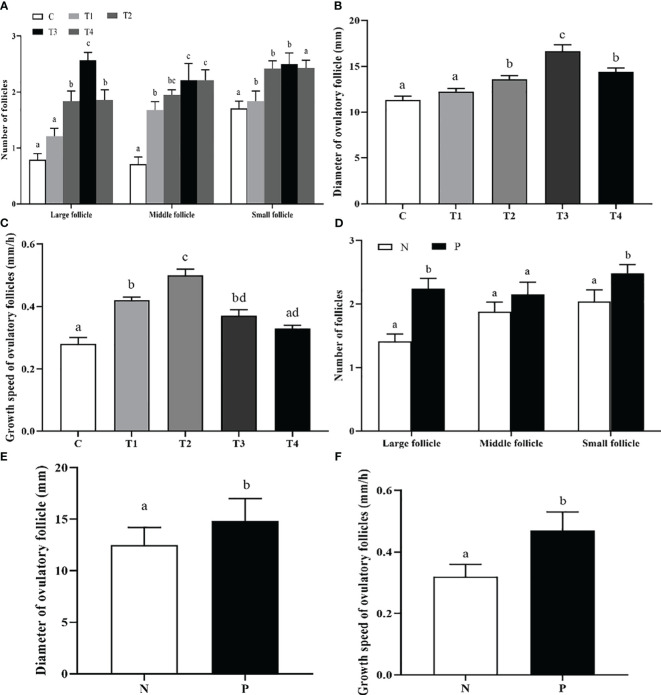
The effect of INH DNA vaccine on follicle counts **(A, D)**, diameter **(B, E)** and growth speed **(C, F)** of ovulatory follicles in beef cattle. Data are presented as mean ± SEM. The bars with different lower case letters indicate significant differences *p* < 0.05 among groups.

Daily ultrasonography was performed to scan and record the number of follicles with different sizes, the diameter and growth speed of ovulatory follicles. As depicted in **Figure 3A**, the large follicles in T2, T3, and T4 groups had a significantly greater number compared to the C group, and greatest count was detected in T3 group (*p* < 0.05); The number of middle follicles in T1, T2, T3, and T4 groups was greater than that of C group (*p* < 0.05); The number of small follicles in T1, T2, and T3 groups was greater than that of C and T4 group (*p* < 0.05). The beef cattle in T2, T3, and T4 groups had a significantly greater diameter of ovulatory follicle compared to the C group (*p* < 0.05), and T3 group was significant greater than other groups (*p* < 0.05) (**Figure 3B**). The growth speed of ovulatory follicle in T1, T2, and T3 groups was much faster compared to C group (*p* < 0.05), and T2 group was significantly greater than other groups (*p* < 0.05) (**Figure 3C**). Furthermore, there was a significant increase of the large and small follicle counts in the P group compared to the N group (*p* < 0.05) (**Figure 3D**). The diameter and growth speed of ovulatory follicle in P group were significantly increased compared with those in N group (*p* < 0.05) (**Figures 3E, F**).

Moreover, we also assessed the percentage (proportion) of beef cattle with different ovulatory follicle counts after immunization with different vaccine concentrations. As presented in **Table 2**, the beef cattle with 2 ovulatory follicles in T2, T3, and T4 groups were significantly increased compared with C group (*p* < 0.05). The beef cattle with 2 ovulatory follicles in the P group were significantly increased compared to the N group (*p* < 0.05) (**Table 3**).

**Table 2:** Percentage (proportion) of beef cattle with different ovulatory follicle counts after immunization with different vaccine concentrations.

Group	0	1	2
C	21.43%(3/14)^a^	78.57%(11/14)^A^	0%(0/14)^A^
T_1_	0%(0/39)^b^	41.03%(16/39)^AB^	58.97%(23/39)^AB^
T_2_	0%(0/40)^b^	25.00%(10/40)^B^	75.00%(30/40)^B^
T_3_	0%(0/26)^b^	15.38%(4/26)^B^	84.62%(22/26)^B^
T_4_	0%(0/30)^b^	20.00%(6/30)^B^	80.00%(24/30)^B^

The different superscript capital and lower case letters in the same column indicate a significant difference p<0.01 and p<0.05 respectively among groups.

**Table 3:** Percentage (proportion) of beef cattle with different ovulatory follicle counts in antibody-positive and antibody-negative group.

Group	0	1	2
P	0%(0/37)^a^	10.81%(4/37)^b^	89.19%(33/37)^b^
N	0%(0/31)^a^	45.16%(11/31)^a^	54.84%(17/31)^a^

The different superscript lower case letters in the same column indicate a significant difference p<0.05 among groups.

### 3.4 Effect of INH DNA vaccine on estrous, ovulation, and conception rates in beef cattle

As presented in **Table 4**, the proportion of ovulation in T2, T3, and T4 groups was significantly superior to that of the C group by 16.30%, 9.33%, and 6.25%, respectively (*p*<0.05). In contrast to C group, oestrus and conception rates were remarkably increased (*p*<0.05) in the immunization groups except for the T3 group. On average, estrous, ovulation, and conception were greater in the antibody-positive beef cattle than those in the negative ones (*p*<0.05) (**Table 5**).

**Table 4:** Rates of estrous, ovulation, and conception after immunization with different vaccine concentrations.

Item	C	T_1_	T_2_	T_3_	T_4_
Oestrus (%)	66.67^a^ (8/12)	69.23^b^ (18/26)	86.96^b^ (20/23)	61.11^a^ (11/18)	75.00^b^ (12/16)
Ovulation (%)	75.00^a^ (9/12)	76.92^a^ (20/26)	91.30^b^ (21/23)	83.33^b^ (15/18)	81.25^b^ (13/16)
Conception (%)	58.33^a^ (7/12)	65.38^b^ (17/26)	78.26^b^ (18/23)	61.11^a^ (11/18)	68.75^b^ (11/16)

The different superscript lower case letters in the same column indicate a significant difference p<0.05 among groups.

**Table 5:** Rates of estrous, ovulation, and conception in antibody-positive and antibody-negative group.

Group	Oestrus (%)	Ovulation (%)	Conception (%)
P	75.51%(37/49)^b^	81.63%(40/49)^b^	69.39%(34/49)^b^
N	52.94%(18/34)^a^	44.12%(15/34)^a^	58.82%(20/34)^a^

The different superscript lower case letters in the same column indicate a significant difference p<0.05 among groups.

### 3.5 Effect of INH DNA vaccine on embryo counts and twinning rate of beef cattle

**Figure 4:**
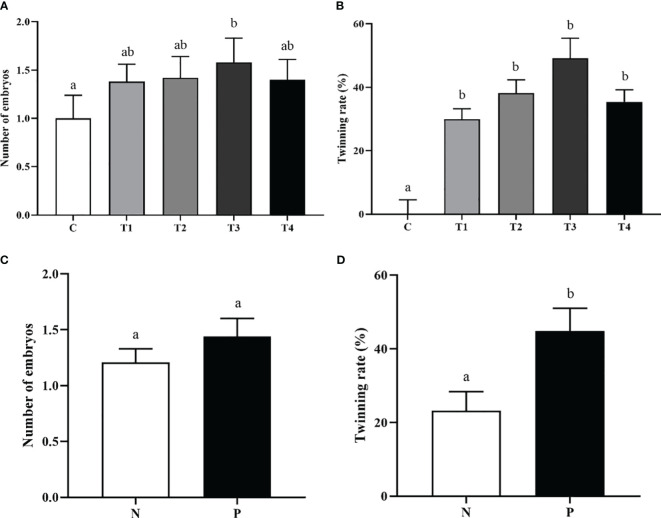
The effect of INH DNA vaccine on embryo counts **(A, C)** and twinning rate **(B, D)** of beef cattle. Data are presented as mean ± SEM. The bars with different lower case letters indicate significant differences *p* < 0.05 among groups.

After immunization with different vaccine concentrations, the embryo numbers in T3 group were notably increased in contrast to C group (*p*<0.05) (**Figure 4A**). The mean twinning rate of beef cattle in T1, T2, T3, and T4 groups was significantly higher than that in C group (*p*<0.05) (**Figure 4B**). However, there was no difference in embryo numbers and twinning rate among T1, T2, T3, and T4 groups (*p*>0.05) (**Figures 4A, B**). In addition, the embryo numbers in P group tended to be higher than the N group, although the difference didn’t reach significance (*p*>0.05) (**Figure 4C**).There was a significant increase of the twinning rate in the P group compared to the N group (*p* < 0.05) (**Figure 4D**).

## 4 Discussion

There is a wealth of evidence that INH recombinant DNA vaccines can improve reproductive function in various animals, but the effect of these DNA vaccines in improving fertility was still inadequate. Both humoral and cellular immune responses could be induced by DNA vaccines (17). Previous studies have demonstrated that some DNA vaccines expressing INH gene have been elicited better immune responses (18). In the current study, the pCISI vaccines evoked the production of anti-INH antibody after both primary and booster immunizations, except for the T1 group on day 10 after primary immunization in beef cattle. In rats, pcISI plasmid containing recombinant statin a subunit (1-32) fragments increased the antibody titers in a dose-dependent manner (19). However, our data showed that the increase in P/N values was not dose dependently varied among treatment groups. We hypothesized that the species differences and dose optimization may have contributed to the bias in the results. Of particular interest was INH immunization, which is generated anti-INH antibody to neutralize the endogenous INH and increase FSH contents, eventually leading to an improvement in reproductive performance (18, 20).

Previous studies about the effect of active immunization with INH on FSH secretion have been inconsistent. Some reports demonstrated that FSH secretions were increased (14, 21), while others found an increase in ovulation rate without an increase in FSH concentrations after active immunization against INH (22, 23). In the present study, we found that the immunized and control groups didn’t show any change in FSH concentration, with the exception of a marked increase of FSH concentration in T2 group on day 45 after booster immunization. These findings suggest that INH may have local autocrine/paracrine effects, one of which was neutralized may augment FSH in enhancing follicular growth in the ovary. Conceivably, the increased number of large follicles observed in treatment groups are responsible for the elevated E_2_ level measured in serum (23, 24).

It has been reported that INH is able to inhibit FSH-induced E_2_ production in granulosa cells (25). E_2_ produced by ovarian follicles plays an important role in governing the estrous cycle and has a direct effect on the reproductive capability of cattle (26). Elevated level of E_2_ and low concentration of P_4_ can promote the estrus expression, follicle maturation and ovulation (27, 28). In our data, the increased estrus and ovulation rate were consistent with higher plasma E_2_ concentrations in the immunization groups. The elevated plasma P_4_ levels in INH immunized heifers reflects the increased ovulation rate and increased number of corpora lutea (4). Similarly, we observed that the concentrations of P_4_ didn’t show any change between N and P groups, except on day 45 after booster immunization. In a practical perspective, our results suggest that the follicles didn’t ovulate and the corpus luteum (CL) formation didn’t occur (14), however the follicle ovulation occurred and the CL was formed on day 45 after booster immunization.

The number of large follicles showed significant increases when mice and rats were immunized with DNA vaccine expressing the INHα (1-32) fragment (18, 29). Furthermore, immunization with INH DNA vaccine enhanced the number of follicles with different diameter sizes, growth speed, and the diameter of the ovulatory follicle, which agree with earlier reports (15). Therefore, the biological activity of endogenous INH is neutralized by active immunization against INH, resulting in increased FSH-induced E_2_ production, thereby stimulating additional or new follicle growth.

Evidence showed that active immunization against INH increases twin-calving and lambing rate (30). Immunization against INH increased the percentage (proportion) of beef cattle with multiple ovulatory follicles in our study. The cattle with multiple ovulatory follicles have an increased probability of multiple ovulations and a higher probability of twinning rate. Previous studies reported that immunization against INH enhanced embryo quality after superovulation and insemination in Holstein heifers (31, 32). The oocyte maturation rate and parthenogenic embryo development potency were increased after treatment with anti-INH α subunit antibodies *in vitro* (33). In the present study, the embryo numbers in T3 group were notably increased, but the DNA vaccines encoding INH genes did not significantly increased in other groups, and there was no significant difference in embryo numbers between antibody-positive and antibody-negative cattle. The reason may be that vaccine-induced antibodies were not enough to completely neutralize endogenous INH, or the number of experiment animals was not sufficient to detect significant increase in embryo numbers of cattle. As well as in other study, beside the increases in total number of embryos, embryo quality was also improved following immunization against INH (34). The mean twinning rate of beef cattle in treatment groups was significantly increased. Such beneficial effects on early embryo qualities and twinning rate following the nullification of INH bioactivities lend us a new hypothesis of improving cattle fertility by immunization against INH.

## 5 Conclusions

This study characterized that neutralization of endogenous INH through intramuscular injection immunization with INH DNA vaccine, successfully elicited a humoral immune response and increased the plasma E_2_ concentration. These findings clearly illustrated that INH DNA vaccine was capable of promoting the follicle development, thereby improving the behavioral of estrous and ovulation, eventually leading to an augment in the conception rates and twinning rate of beef cattle.

## Data availability statement

The original contributions presented in the study are included in the article/supplementary material. Further inquiries can be directed to the corresponding authors.

## Ethics statement

All cattle were treated according to the Ethical Committee of Animal Experiments, Hunan Agricultural University (No. 432022021). The studies were conducted in accordance with the local legislation and institutional requirements. Written informed consent was obtained from the owners for the participation of their animals in this study.

## Author contributions

JM: Formal analysis, Visualization, Writing – original draft. QL: Methodology, Writing – review & editing. LX: Software, Writing – review & editing. WL: Methodology, Writing – review & editing. ZG: Resources, Writing – review & editing. LG: Resources, Writing – review & editing. XL: Visualization, Writing – review & editing. SW: Funding acquisition, Validation, Writing – review & editing.
